# The Cancer Microenvironment: Mechanical Challenges of the Metastatic Cascade

**DOI:** 10.3389/fbioe.2021.625859

**Published:** 2021-02-12

**Authors:** Sebastian E. Amos, Yu Suk Choi

**Affiliations:** School of Human Sciences, The University of Western Australia, Perth, WA, Australia

**Keywords:** extracellular matrix, confinement, mechanotransduction, invasion, biophysics, stiffness

## Abstract

The metastatic cascade presents a significant challenge to patient survival in the fight against cancer. As metastatic cells disseminate and colonize a secondary site, stepwise exposure to microenvironment-specific mechanical stimuli influences and protects successful metastasis. Following cancerous transformation and associated cell recruitment, the tumor microenvironment (TME) becomes a mechanically complex niche, owing to changes in extracellular matrix (ECM) stiffness and architecture. The ECM mechanically reprograms the cancer cell phenotype, priming cells for invasion. 2D and 3D hydrogel-based culture platforms approximate these environmental variables and permit investigations into tumor-dependent shifts in malignancy. Following TME modification, malignant cells must invade the local ECM, driven toward blood, and lymph vessels by sensing biochemical and biophysical gradients. Microfluidic chips recreate cancer-modified ECM tracks, empowering studies into modes of confined motility. Intravasation and extravasation consist of complex cancer-endothelial interactions that modify an otherwise submicron-scale migration. Perfused microfluidic platforms facilitate the physiological culture of endothelial cells and thus enhance the translatability of basic research into metastatic transendothelial migration. These platforms also shed light on the poorly understood circulating tumor cell, which defies adherent cell norms by surviving the shear stress of blood flow and avoiding anoikis. Metastatic cancers possess the plasticity to adapt to new mechanical conditions, permitting their invasiveness, and ensuring their survival against anomalous stimuli. Here, we review the cellular mechanics of metastasis in the context of current *in vitro* approaches. Advances that further expose the mechanisms underpinning the phenotypic fluidity of metastatic cancers remain central to the development of novel interventions targeting cancer.

## Introduction

Cancer remains a leading cause of death globally; a burden largely attributed to cancer cell metastasis (World Health Organisation, 2018). While the genetic and biochemical drivers of metastasis are widely recognized, biophysical stimuli also progress cancer. Like all other cell types, cancerous cells perceive physical inputs from their microenvironment that mechanically alter DNA transcription and, thus, cell behavior and function, a process known as mechanotransduction (Eyckmans et al., 2011; Broders-Bondon et al., 2018). Such stimuli include the stiffness, composition, and architecture of the extracellular matrix (ECM), the mechanotransduction of which enhances and protects successful metastasis (Wei et al., 2015). All stages of the metastatic cascade are inherently mechanical, as cells invade through and interact with tissues and fluids of varying compositions and rheological properties. While these stimuli are implicated in malignancy, their contributions remain incompletely understood. Our ability to study metastatic mechanics *in vitro* is dependent on microfluidics and biomimetic substrates. Ongoing developments in these platforms continue to refine *in vitro* investigations of the cancer-microenvironment interface. Here, we review key biophysical mechanics of the metastatic cascade and our ability to study them *in vitro* to further our understandings of this complex disease.

## The Tumor Microenvironment

Cancerous transformation inflames the surrounding tissue and activates cancer-associated cell types, prompting the pathogenesis of the tumor microenvironment (TME) (Hanahan and Weinberg, 2011; Kim and Bae, 2016; Yamauchi et al., 2020). This is characterized by the upregulated deposition, reorganization, and increased crosslinking of ECM proteins, such as fibronectin and collagen type I (Tschumperlin and Lagares, 2020). This disruption of ECM homeostasis alters matrix deformability and ligand availability, thus perturbing local cell mechanotransduction. Moreover, increased ECM deposition and crosslinking compartmentalizes and compresses the tumor as its diverse cellular population proliferates (Tse et al., 2012; Vennin et al., 2018). The destabilized mechanical and biochemical profiles of the TME coalesce to drive pre-metastatic phenomena, such as epithelial-mesenchymal plasticity (EMP) (Redfern et al., 2019). In isolating TME mechanics’ effect on tumor progression and metastasis, investigators employ a combination of 2D and 3D hydrogel-based cell culture systems. The stiffness, composition, and pore size of the ECM can be highly controlled and manipulated within such hydrogel platforms, granting control over the important haptotactic and durotactic stimuli that drive metastasis (Table 1).

**TABLE 1:** The advantages and disadvantages of selected platforms to study the mechanics of the metastatic cascade.

*Metastatic cascade*	*Platform*	*Description*	*Advantages*	*Disadvantages*	*Key papers*
***Tumor Microenvironment***	2D Nano-spacing	Block copolymer micelle nanolithography (BCMN) and peptidomimetics are used to synthesize nano-spaced peptide-coated particles on a culture substrate	ECM ligand density can be highly controlled Single-cell resolution	2D cultures do not recreate 3D *in vivo* cell-cell and cell-ECM interactions	Young et al., 2020 Amschler et al., 2018
	3D Hydrogels	Tuneable semi-synthetic hydrogels such as GelMA and alginate-based interpenetrating networks utilize UV or Ca^2+^ crosslinking to modulate substrate stiffness (and pore size in GelMA)	Replicates cell-cell and cell-ECM interactions On-demand (temporally and spatially) tuneable stiffness/pore size Elastic and viscoelastic options	Reduced imaging quality/ease of imaging with increasing sample thickness Unable to replicate the diversity of natural ECM	Panciera et al., 2020 Joyce et al., 2018 Kim C. et al., 2020 Peela et al., 2016
***Invasion***	3D Hydrogels	Tuneable natural hydrogels such as collagen type I or reconstituted basement membrane are thermally polymerized. Substrate stiffness can be controlled by adjusting protein concentration and gelation temperature	Tuneable soft stiffness’s Native ECM proteins Viscoelastic properties close to *in vivo* conditions	Tuneable stiffness typically does not cover the complete physiological range Cannot control pore size	Chaudhuri et al., 2014 Wullkopf et al., 2018
	Microchannels	Soft lithography is used to fabricate microchannels of varying dimensions and topographies by casting polydimethylsiloxane over silicon wafers/molds	High spatial resolution Relatively cheap Routine microscopy compatible	Reduced substrate stiffness tuneability Unable to recreate true heterogeneity of tissue topography	Holle et al., 2019 Ma et al., 2018 Microchannels created in collagen address this, see Mosier et al., 2019
***Intra/Extravasation***	Co-culture Microfluidics	Soft lithography is used to fabricate perfused microfluidic chips designed to accommodate different cells types that can communicate and interact through media or hydrogel reservoirs	Physiological culture of endothelial cells in platforms with flow Inter-cellular communication	Increased cost, preparation time, and resource demand Reduced data resolution with increasing complexity	Chen et al., 2013 Nguyen et al., 2019
	Subnuclear Microchannels	Soft lithography or glass etching allows for the fabrication of subnuclear constriction challenges. Nuclear constriction topographies include periodic pinch-points and restricted channels	Highly controlled constriction dimensions Single-cell resolution with manipulability	Limited control of perceived substrate stiffness Increased preparation time and required resources	Raab et al., 2016 Sima et al., 2020
***Circulating Tumor Cells***	Microfluidics	Soft lithography-fabricated microfluidic chips are connected to pumps that circulate cell media and thus exert shear stresses on cells and/or maintain them in suspended culture	Application and control of fluidic shear stress Reduced handling during experimentation	Lacking interaction with native blood/lymph cells Live-cell imaging resources Increased preparation time	Zhang et al., 2014 Fan et al., 2016 Can be utilized for real-time deformability cytometry see Otto et al., 2015
	Metastasis-on-a-chip	Composite platforms incorporating a combination of the above platforms (i.e., 3D encapsulated cell types and perfused microfluidics) to study the metastatic cascade in an integrated fashion	Incorporation of many *in vivo* variables Multi-system chip scalability	Reduced data resolution with increasing complexity Optimizing culture media Increased preparation time and required resources Low through-put	Rajan et al., 2020b Aleman and Skardal, 2019 Hassell et al., 2017

### Extracellular Matrix Stiffness

Perhaps one of the most well documented solid TME characteristics is associated ECM stiffening. While this is downstream of initial tumorigenesis, owing to the recruitment and activation of cancer-associated fibroblasts, there is an established link between ECM stiffening and metastasis. 2D and 3D (encapsulating) hydrogel platforms (such as collagen, gelatin, or alginate-based gels) find that increased ECM stiffness drives invasion in metastatic breast cancer cells, while non-cancerous cells did not exhibit such invasive phenotypes (Levental et al., 2009; Chaudhuri et al., 2014; Peela et al., 2016; Ondeck et al., 2019). This may owe to oncogene-mediated changes in mechanosensitivity, which alters the transduction of ECM stiffening (Panciera et al., 2020). While this mechanoperception, at least in part, utilizes established mechanosensitive transcriptional regulators YAP/TAZ, 3D encapsulation reduces cross-sectional force exposure, suggesting metastatic mechanosensation may operate through parallel, YAP-independent pathways (Lee et al., 2019). This reprogramming of mechanosensation further enhances phenotypic plasticity, whereby the viscoelasticity of metastatic cells is dynamic and environmentally impressionable compared to their non-metastatic counterparts (Tian et al., 2020). Interestingly, stiffness-dependent chemoresistance is also observed in 2D and 3D hydrogel platforms (Rice et al., 2017; Joyce et al., 2018). Moreover, this stiffness-mediated resistance was only observed in metastatic cell lines, suggesting that phenotypic plasticity and prosurvival activation in metastatic cancer cells are both mechanically coupled.

### Extracellular Matrix Architecture

**FIGURE 1:**
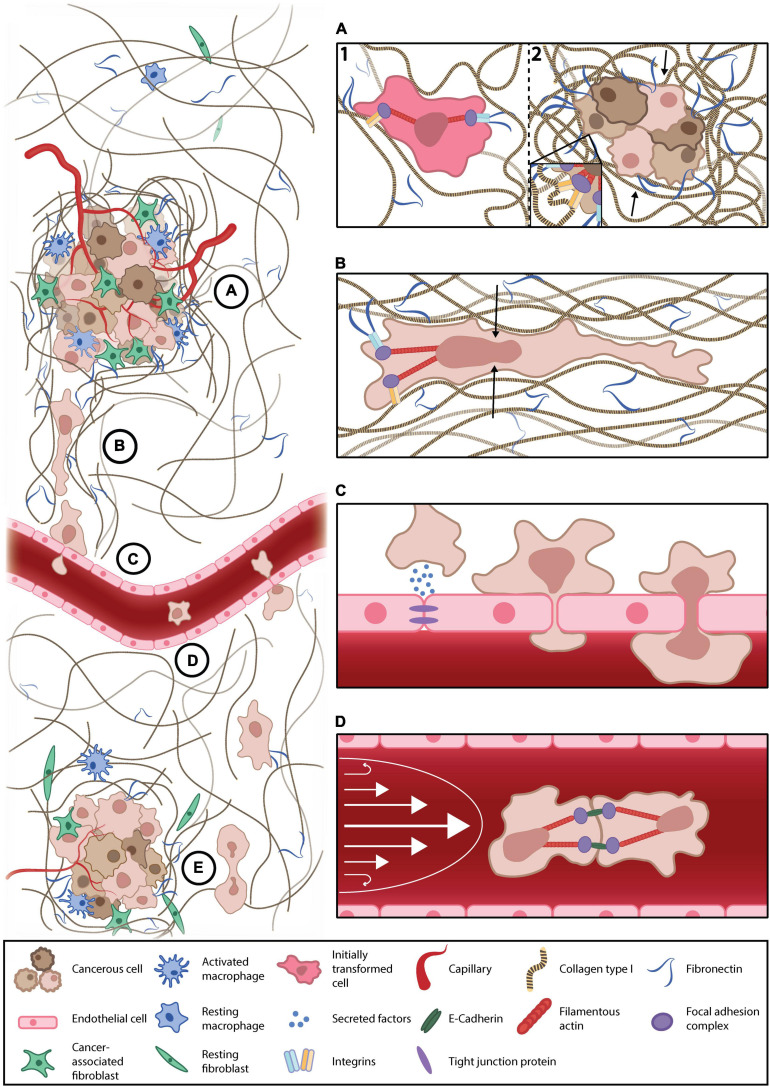
Key mechanical forces of the metastatic microenvironment. **(A)** Cancer-associated remodeling of the normal extracellular matrix (ECM) (1) increases local stiffness and alters ligand availability in and around the tumor microenvironment (TME), the mechanotransduction of which enhances cancer cell survival, proliferation, and primes cells for metastasis (2). **(B)** Invasive cells escape the primary tumor through confined, subnuclear ECM tracks. Such confinement deforms the nucleus (black arrows) and reprograms anchorage dependency, thereby altering transcriptional regulator translocation, conventional mechanotransduction pathways, and, thus, the invasive phenotype. **(C)** Current understandings of transendothelial migration suggest interacting cancer cells mechanically and chemically modify these submicron constrictions with permeabilizing factors and cancer-associated inflammation of the endothelium. **(D)** Cancer cells must survive anoikis while disseminating in the blood/lymph. They may evade anoikis by clustering to engage cell-cell adhesions that generate intracellular tension that is transduced to the nucleus, replacing the lost input of substrate adhesion, thereby suppressing anchorage-dependent apoptosis. Moreover, suspended cancer cells (or circulating tumor cells) must survive the shear stress of blood flow (white arrows). **(E)** Upon reaching a distant secondary site, metastatic cells must survive in a foreign environment, the mechanical and chemical profiles of which differ from their tissue of origin.

In addition to stiffening, cancer-associated ECM is more dense and aligned, forming ECM highways for invading cells and altering ligand spacing within and around the tumor. Changes in ligand availability alter integrin subunit involvement, clustering, and focal adhesion complex assembly, thus perturbing intracellular signaling cascades that influence cell behaviors, including migration, proliferation, and survival (Figure 1A; Levental et al., 2009; Jang and Beningo, 2019). As such, when interacting with a sparse, non-cancerous ECM, the invasive and proliferative tendencies of readily metastatic cells are suppressed, suggestive of a ligand-dependent reprogramming that is maintained with a change in microenvironment (Kaukonen et al., 2016). While normal and cancerous fibroblast-generated matrices demonstrate the importance of ligand density, these techniques cannot be well controlled, nor their variables (such as compounded stiffness) isolated. The advent of 2D nano-spaced ligand platforms permits the investigation of ECM density with single-cell resolution. Studies find that cancer cell morphology, motility, plasticity, and adhesion are manipulated in a ligand density-dependent manner (Lee et al., 2011; Amschler et al., 2014, 2018; Horzum et al., 2015). Interestingly, varying ligand density demonstrates a proportional exchange between cell-cell and cell-ECM adhesion (Horzum et al., 2015). Moreover, cells on controlled ligand spacing platforms have also exhibited shifts in states of EMP, implicating ECM density in metastatic progression (Marlar et al., 2016). These nano-spaced ligand platforms have recently been combined with blocking peptidomimetics to delineate integrin subtype involvement in breast cancer drug resistance. Young et al. (2020) demonstrated that metastatic breast cancer drug sensitivity was highly dependent on ligand spacing and integrin subtype, thus affirming ECM architecture’s influence on metastatic protection and progression. Finally, regarding *in vitro* models of the TME, the importance of co-culture platforms, through which ECM, phenotypic, and chemoreceptive norms are modified by accessory cell types, such as cancer-associated fibroblasts and macrophages, must also be acknowledged (Kuen et al., 2017; Plaster et al., 2019; Vennin et al., 2019; Huang et al., 2020; Libring et al., 2020; Lugo-Cintrón et al., 2020).

## Invasion in the Local Microenvironment

To escape the TME, disseminating cells must invade toward intra- or extra-tumoral blood and lymph vessels (Nguyen et al., 2009). ECM crosslinking and the consequently decreased pore size and rate-limiting factors such as nuclear size and integrity mechanically govern cancer cell invasion (Wisdom et al., 2018). Tumor-modified ECM tracks are narrower than 5 microns in some instances, presenting invasive cells with prolonged and repeated constriction challenges (Weigelin et al., 2012). Such confinement differentially influences an invading cell’s motility and resilience, depending on their metastatic competence, which owes to TME reprogramming (Bhatia et al., 2020). Polydimethylsiloxane (PDMS)-cast microchannel devices permit the mimicry of narrowed ECM pores/tracks with precisely controlled channel dimensions. These platforms endeavor to highlight confinement-dependent changes in metastatic patterns of locomotion.

### Mechanical Confinement Enhances Cancer Invasion

Recent studies demonstrate that cancer invasion speed increases with the degree of constriction in long, representative microtracks (Holle et al., 2019; Mosier et al., 2019; Wang et al., 2019). Interestingly, invasion speed also increases with the number of brief, periodic constriction challenges (Mak et al., 2013; Ma et al., 2018). These studies observe confinement-dependent motility changes, with some reporting a mesenchymal to amoeboid-type shift in locomotion, owing to an adhesion-independent reprogramming, as is observed in immune cell invasion (Reversat et al., 2020). This reprogrammed adhesion has been recently highlighted and is accompanied by cell softening consistent with decreases in force exertion on the surrounding ECM (Guck et al., 2005; Kristal-Muscal et al., 2013; Khan et al., 2018; Holenstein et al., 2019; Beri et al., 2020; Han et al., 2020). Importantly, these mechanical traits of an amoeboid phenotype are evident in patient samples (Swaminathan et al., 2011; Plodinec et al., 2012). These data assert an amoeboid transition under confinement as a distinct paradigm during cancer invasion that traditional models of epithelial-mesenchymal transition are unable to characterize.

Nuclear membranes are coupled to the ECM through cytoskeletal fibers in established mechanotransduction pathways (Holle et al., 2018). In amoeboid-transformed cells, reduced ECM coupling obscures classical models of mechanotransduction. However, mechanically gated nuclear pores stretch during nuclear deformation, promoting the shuttling of transcriptional regulators, and activating signaling cascades that modify cell migration and behavior (Elosegui-Artola et al., 2017; Venturini et al., 2020). Under extreme constriction, the nuclear envelope ruptures, resulting in the mixing of the cytosolic, and nuclear contents; a phenomenon that does not impede cancer invasion (Denais et al., 2016; Raab et al., 2016). Interestingly, metastatic cells become more invasive following nuclear envelope rupture, whereas non-cancerous cells undergo accelerated senescence (Nader et al., 2020). Therefore, a deformed or repeatedly ruptured nucleus under confinement may become an independently mechanosensitive apparatus responsible for guiding cell behavior during cancer invasion (Figure 1B). However, the role of such nuclear mechanics in cancer invasion remains a current topic of interest (Fu et al., 2012; Kirby and Lammerding, 2018; Mierke, 2019; Heo et al., 2020; Herráez-Aguilar et al., 2020; Lomakin et al., 2020).

## Intravasation and Extravasation

The reprogramming of invasive cells into less matrix-dependent, amoeboid-like cells demonstrates the phenotypic plasticity of metastatic cells but likely functions in preparing invading cells for the extreme confinement encountered during intravasation and extravasation (Chiang et al., 2016). Upon reaching an intra- or extra-tumoral vessel, invasive cells must traverse the endothelial barrier, which is bound by submicron-scale cell-cell adhesions, including tight junctions (Wallez and Huber, 2008). Invasive cancer cells and associated cell types, such as macrophages, may modify these junctions to reduce the degree of confinement experienced, although these mechanisms are not fully known (Zervantonakis et al., 2012). Researchers integrate microfluidic PDMS devices with biomimetic substrates (hydrogels) and multicellular co-cultures to interrogate cancer-endothelial mechanics. Similar to studies of invasion, the extreme confinement of *trans-*endothelial migration can be approximated using advanced microfabrication techniques.

### Microfluidic Co-culture Platforms Reveal Complex Cancer-Vessel Interactions

Perfused microfluidic devices recreate one of the most formidable mechanical stresses of the metastatic cascade, fluidic shear stress. These platforms facilitate the physiological culture of endothelial cells, the permeability, and morphology of which are mechanically regulated by flow, thus, improving research translatability of endothelial traversal or junction modification (Wang et al., 2013; Sfriso et al., 2018). Perfused platforms demonstrate that both flow rate and pulsatility influence cancer-endothelial adhesion and subsequent traversal (Kühlbach et al., 2018). Furthermore, *trans-*endothelial migration is cooperated by disruptions in endothelial permeability owing to external mechanical and chemical perturbation; as is observed in cancer-associated macrophage activation or exposure to tissue-specific factors (Figure 1C; Jeon et al., 2013, 2015; Lee et al., 2014; Peng et al., 2019; Zavyalova et al., 2019). Not all cancer cells possess the ability to traverse the endothelium, which may reflect the phenotypic heterogeneity of metastatic cells (Jeon et al., 2013; Bertulli et al., 2018). Interestingly, mechanically resilient amoeboid phenotypes have been observed during intravasation, evidenced by macrophage-mediated RhoA activity (Kosla et al., 2013; Roh-Johnson et al., 2014). An important study by Chen et al. (2013) demonstrates the endothelium’s pliability as a mechanical barrier, visualizing increases in endothelial apertures throughout a single extravasation. They also report clusters of extravasating cells, which may increase local endothelial exposure to permeabilizing, proinflammatory cancer secretions, further destabilizing the endothelium and enhancing metastatic progression (Chen et al., 2013). In platforms that lack perfusion, metastatic cells still perturb the endothelium’s structural integrity to facilitate intravasation, findings that are supported *in vivo* (Nguyen et al., 2019).

While co-culture studies more accurately recapitulate cancer-endothelial interactions, they make it challenging to isolate the submicron constriction mechanics that may constitute intra- and extravasation. Recently, glass microfluidic devices with submicron constriction challenges were fabricated with femtosecond laser-assisted etching. While this platform does not recreate other essential variables, such as ECM stiffness, it demonstrates that metastatic cells are capable of submicron invasion and that, as previously reported, invasion speeds increase with constriction. Crucially, this mechanical challenge did not impair post-constriction proliferation or migration (Sima et al., 2020).

## Surviving in Suspension

Cancer cells can escape many forms of programmed cell death through a myriad of signaling cascades, some of which are mechanically coupled (Hanahan and Weinberg, 2011). Once invading cells have successfully intravasated, they must survive hemodynamic shear stresses and escape anchorage-dependent apoptosis, known as anoikis (Paoli et al., 2013). In suspension, an adherent cell should undergo anoikis owing to the loss of integrin-mediated apoptotic suppression, as is observed in normal epithelial turnover (Frisch and Screaton, 2001; Paoli et al., 2013). Nevertheless, a suspended cancer cell can withstand this loss of mechanical signaling and disseminate as a circulating tumor cell (CTC). The mechanisms through which CTCs evade anoikis are conflicting, but have been recently eluded to in studies employing microfluidic systems. As with studies of intra- and extravasation, perfused microfluidic devices provide an approximated physiological mimic within which the behaviors of CTCs may be investigated. Combining these perfused devices, with conditioned CTCs and other, hydrogel-embedded cell types grants an unprecedented look at the metastatic cascade in its entirety; an emerging study tool known as metastasis-on-a-chip.

### Circulating Tumor Cells

Circulating tumor cells form multicellular clusters *in vitro* and *in vivo* (Chen et al., 2013; Yu et al., 2013; Aceto et al., 2014; Au et al., 2016). This cell-cell adhesion, and subsequent engagement of adherens junction proteins, such as cadherin, initiates mechanically coupled antiapoptotic signaling cascades and thus, afford CTCs time to disseminate in suspension (Guadamillas et al., 2011; Li et al., 2019; Tang et al., 2020). Studies demonstrate that the mechanotransduction of shear stress may facilitate this phenotypic shift (Zhao et al., 2014; Yang et al., 2016; Follain et al., 2020). Moreover, cancer cells exposed to physiological shear stress are more invasive, proliferative, and chemoresistant than non-cancerous cells and CTCs in static conditions (Lee et al., 2017, 2018; Novak et al., 2019). Therefore, not only are CTCs resistant to anoikis and physiological shear stresses, but such stimuli potentiate metastasis (Figure 1D; Barnes et al., 2012; Zhang et al., 2018). CTC clusters remain highly deformable while maintaining cell-cell adhesions, permitting the navigation of capillary-sized constrictions (Au et al., 2016). Shear stress also enhances extravasation and migration in CTCs, owing to increases in cellular oxidative stress (Ma et al., 2017). Interestingly, while initially softer, metastatic cells stiffen following shear stress exposure, while their non-cancerous counterparts were unresponsive. This reinforces an oncogene-mediated reprogramming of cellular mechanosensitivity and cytoskeletal mechanoadaptation (Chivukula et al., 2015).

### Metastasis-on-a-Chip

Throughout the metastatic cascade, one mechanical exposure seemingly prepares the invading cell for the next. Logically, this progression should be studied in an integrated fashion, rather than in isolation, as is traditional of reductionist research. As such, some metastasis-on-a-chip platforms allow researchers to study each stage of the metastatic cascade in a single microfluidic device (Sleeboom et al., 2018; Sontheimer-Phelps et al., 2019). These facilitate investigations into metastatic enigmata, like organotropism, whereby metastasizing cells have a secondary tissue preference (Figure 1E; Hoshino et al., 2015). While in its infancy, organotropic studies of metastasis do elude to disseminative preference and demonstrate stiffness-dependent TME escape, reflecting *in vivo* observations (Skardal et al., 2016; Aleman and Skardal, 2019). While cellular mechanics become difficult to resolve with increasing system complexity, important physical cues may be reproduced, and their effects on cancer progression, examined; such as the cyclic tension of respiration in a model of lung metastasis (Hassell et al., 2017). Such biomimetic systems also lend themselves to pharmacological and biochemical screening, granting researchers insight into how such conditioning modifies and influences the biophysics of metastatic microenvironments (Kim J. et al., 2020; Nashimoto et al., 2020; Palacio-Castaneda et al., 2020; Rajan et al., 2020a, b; Sharifi et al., 2020).

## Conclusion and Future Perspectives

Recent research establishes the stepwise biophysical cues of the metastatic cascade as essential drivers of malignancy. Each step of the metastatic cascade presents an opportunity to perturb cancer’s mechanically coupled progression. Unfortunately, the mechanisms that underlie the influence of a cancer cell’s microenvironment on its remarkable plasticity and resilience remain incompletely characterized. Ongoing developments in bioengineering promise to advance our capacity to resolve single-cell level changes in response to microenvironment-specific mechanics. While such resolution will surely highlight new therapeutic targets that underlie the burden of metastatic cancer, these studies are principally conducted with immortal, commercially available cell lines. While these cell lines have informed cancer biology over many decades, they do not wholly mimic the phenotypic plasticity or responsiveness observed *in vivo*. Phenotype and behavior may vary more than can be captured by currently available cell lines, thus, idealizing the development of more native, dynamic alternatives. Such developments would further empower metastasis-on-a-chip platforms, facilitating more physiological investigations of the cell-microenvironment interface. Moreover, the prospect of mechanotherapy, such as reversing the ECM remodeling of the TME and surrounding stroma, may prove a beneficial adjunct therapy by improving the efficacy of chemotherapy, thus bettering patient outcomes (Vennin et al., 2018; Tschumperlin and Lagares, 2020). In conducting such research, investigators must acknowledge the mechanosensitivity of metastatic cancers and the mechanical profiles that constitute the metastatic cascade. Here, we highlight the cellular responses to key microenvironmental stimuli that corroborate metastasis and represent future therapeutic targets.

## Author Contributions

SA reviewed the literature, prepared the figure, and wrote the manuscript. YC edited and supervised the manuscript. Both authors approved the manuscript for publication.

## Conflict of Interest

The authors declare that the research was conducted in the absence of any commercial or financial relationships that could be construed as a potential conflict of interest.
